# How voluntary control over information and body movements determines “what it’s like” to have perceptual, bodily, emotional and mental experiences

**DOI:** 10.3389/fpsyg.2022.1108279

**Published:** 2023-01-17

**Authors:** J. Kevin O'Regan

**Affiliations:** Integrative Neurosciences and Cognition Center, CNRS, Planet Learning Institute, Université Paris Cité, Paris, France

**Keywords:** phenomenal consciousness, hard problem, what it’s like, sensorimotor theory, voluntary control, body movements, perceptual presence, locus of attribution

## Abstract

Two very fundamental aspects of phenomenal experiences underline the fact that they seem to have “something it’s like.” One aspect is the fact that experiences have a locus: they Can seem “external” (perceptual), “internal” (interoceptive, bodily or emotional) or “mental.” A second fundamental aspect is the imposingness of experiences. They can seem “present” to us in different ways, sometimes seeming displayed before us with “spatio-temporal presence.” Both these aspects of “what it’s like” can be identified with the degree to which we can voluntarily control what we are doing when we engage in an experience. The external/internal/mental dimension is determined by how our voluntary bodily actions can influence the sensorimotor flow of information. The degree of imposingness of experiences and their “spatio-temporal presence” Is determined by how our voluntary actions are impeded or assisted by innate, attention-grabbing mechanisms. By elucidating these two most fundamental aspects of “what it’s like,” and taken together with prior work on inter- and intra-modal differences in experiences, this article suggests a path toward a scientific theory of the “hard problem” of phenomenal consciousness, explaining why experiences feel like something rather than feeling like nothing.

## 1. Introduction

A characteristic of experiences that is often referred to in the philosophical literature is the fact that people tend to agree that “there’s something it’s like” rather than that there is “nothing it’s like” to have an experience (*cf.* Farrell, 2016, for a critical review). To make progress in overcoming this “hard problem” (Chalmers, 1995) and providing a scientific account of this “something it’s like,” it seems necessary to ask what might actually be meant by the statement that there is “something it’s like.”

One contribution to what might be meant is the fact that when people are having phenomenal experiences, they as persons are poised to be able to report upon the nature of the experience, or to be able to modify their decisions or plans or behavior as a function of the experience. This aspect of the meaning of “what it’s like” to be experiencing can be related to Block’s (1996) notion of “conscious access”.^1^ It is an aspect that is amenable to scientific investigation, with numerous laboratories currently investigating the neural, cognitive and attentional mechanisms that underlie it (*cf.* Doerig et al., 2020).

Another contribution to what might be meant by saying that experiences have “something it’s like” could be the fact that they are subjective, that is they are “for-me,” and furthermore appear unified and “presentational” to me and have properties like being first person and immune to error through misidentification (for surveys see e.g., Prosser and Recanati, 2012; Williford et al., 2012). This aspect of the meaning of “what it’s like” is intimately related to the notion of “self.” As such it seems reasonable to hope that these “subjective,” self-related properties of experience may be given an explanation in terms of the cognitive, attentional and also social functions of the self and how it is involved in the exploratory, probing, activity that experiencing consists in.

Finally, another contribution to what might be meant by saying that experiences have “something it’s like” is related to the fundamentally “phenomenal” aspect of experiences, namely the fact that experiences have qualities. This statement can on one hand be interpreted as referring to the fact that experiences can be distinguished from one another. For example in the case of sensory experiences one can make distinctions between modalities (e.g., distinguish vision from audition or distinguish vision from touch), or one can make distinctions within the modalities themselves (e.g., distinguish red from pink, or high-pitched from low-pitched).^2^ In the case of emotions one can distinguish different types (e.g., basic emotions like fear, anger, sadness, secondary emotions like shame, relief and guilt), and within each type one can also find subtle differences. Similar distinctions can be made between and within varieties of bodily and mental experiences.

On the other hand the statement that experiences have qualities can also be interpreted as emphasizing the fact that experiences have qualities *rather than no qualities at all.* What exactly might be meant by that? I suggest that perhaps what might be meant is not only that experiences can generally be reported (as in the notion of conscious access); and not only that experiences are experienced by selves and occur to us and for us; and not only that there are differences between different experiences but additionally that: First, experiences have a locus of attribution -- they impinge upon us from the world, the body or the mind. And second, experiences are perceptually “present” to us in different degrees and ways -- they impose themselves on us to different degrees. I suggest that these two aspects of experiences may be important additional contributors to what people are referring to when they say that experiences have “something it’s like” rather than “nothing it’s like at all.” I suggest that the very profound feeling we have that experiences are “phenomenal,” i.e., that they intimately affect us, could be rooted in the two facts that they impinge on us from the world, our bodies or our minds, and that they impose themselves on us in certain ways.

In the present article I show how these two very fundamental subjective aspects of experiences can be accounted for in terms of **objective** facts about our ability to control what we are doing when we have experiences. More precisely I show first that the worldly, bodily, or mental locus of attribution of an experience can be identified with the degree to which we can use our body movements to control the sensorimotor flow of information. Second I show that the degree and nature of the perceptual “presence” of an experience can be identified with the degree and way in which sensory events can make us lose control of the exploratory activity that constitutes experiencing.

The approach is part of the sensorimotor approach to understanding phenomenal consciousness (O’Regan, 2010, 2011, 2012, 2014). It is what I have called an “analytic phenomenology” (O’Regan et al., 2004) that consists in analyzing and decomposing into scientifically tractable parts what people **mean** when they consider themselves to be having phenomenal experiences. I suggest that what people mean is that they as persons (or selves^3^) are implicitly engaged in mentally noting and probing the actual or potential sensorimotor laws that currently govern their interaction the world. The approach is empirical and scientific, for example explaining inter- and intra-modal quality differences and stimulating research on topics like change blindness, sensory substitution, illusions of ownership, color naming, pain, and dissociative disorders. Because the approach relies on everyday-language descriptions of sensorimotor interactions to explain phenomenology, it avoids the “explanatory gap” between the language of experience and the language of neural mechanisms (Levine, 1983). As a consequence the approach does not need to appeal to arcane cortical mechanisms like quantum gravity effects in microtubules, information integration or cortico-thalamic reverberations, that seem to have no intelligible link with the phenomenal qualities of experiences.

Note that the analysis presented in the next sections should be considered tentative and open to debate. The purpose is not to give a definitive account of “what it’s like,” but to show that the sensorimotor approach provides a method by which “what it’s like” can be scientifically addressed in terms of sensorimotor laws. Details must be fleshed out and errors corrected using more psychophysical expertise. Further work may also reveal whether neuro-anatomic connectivity supports the claims made here about the way different types of experiences are wired to interfere with voluntary control.

## 2. The external/internal/mental dimension of experience and control over information changes caused by voluntary body actions

As explained in the introduction, the present article suggests that two aspects of experience, namely “locus” and “imposingness,” may contribute fundamentally to people’s impression that experiences have “something it’s like” rather than “nothing it’s like.” This section is concerned with “locus.”

Some experiences (like seeing and hearing) generally have an external quality, seeming to originate in the world and to impinge on us from outside. Some experiences have a more bodily character and seem to occur inside of us (thirst, hunger, and pain). Some experiences affect us both bodily and “as a person” (anger, sadness, and shame). And some experiences seem to be purely mental, with no precise physical location (feeling confused, hopeful, and ridiculous). Additionally there may also be an ongoing vs. state-like dimension of experience related to whether the experience appears to be “occurring to us,” versus whether it is more static, like a “state” or “disposition.” For example hearing a siren seems to be a thing that impacts us perceptually in an ongoing fashion over time, contrary to perceiving our balance -- balance is more state-like and does not impact us in a continuous fashion: we do not “perceive” our balance, we just “are” in equilibrium.

Such distinctions about experiences are so obvious that we tend to take them for granted. Yet the objective reason why we make these distinctions is not at all obvious: you might be tempted to say that since perceptual experiences are exteroceptive, providing information from the outside world, they must necessarily be of a perceptual nature, while interoceptive information about the body must be more bodily and state-like, and information about our thoughts and mental dispositions must be mental.

But thinking like this would be begging the question. Why should the physical or neural origin of information determine the way it feels? For example, why should information coming into our brains that originates from the outside world create an impression of being perceptual, i.e., of information impinging on us from outside? After all, the information is just neural activity like any other neural activity in the brain. And why should certain other neural activity, corresponding to interoceptive states, induce a state-like, bodily experience? And why should yet other neural activity give a mental experience?

From the point of view of the sensorimotor approach (O’Regan, 2011), the answer to these questions must be sought in a careful analysis of what we **mean**, or what it **consists in** to have “external,” “internal” or “mind-like” experiences. What is different about what we **do** (or potentially do) when we engage in these different experiences?

So for example, what does having an exteroceptive experience, acquiring information about the outside world **consist in**? What are we **doing** when we are engaged in probing the outside world?

### 2.1. Bodiliness, insubordinateness, and interruptibility in “external” experiences

One obvious property of what we mean by an experience of the “outside world” is that it escapes our control. Thus, one thing we are doing when we are having an experience with the outside world is that we are implicitly noting that the flow of information deriving from our exteroceptors can vary of its own accord, without any action on our part. The incoming information has a “will of its own,” it is insubordinate to our voluntary efforts. I call this property: “insubordinateness.”

But while the sensorimotor interaction characterizing exteriority has this property of insubordinateness, escaping our will, it can nevertheless also be partially subjugated, namely by our body movements. The reason for this is that the signals provided by our exteroceptive sensors are not only dependent on properties of the external source, but generally depend also on the spatial relation between the source and the sensor. When parameters like the angle or the distance between source and sensor change, the signal changes. Since our sensors are mounted on our bodies, this means that we can voluntarily, by moving our bodies, modify the incoming signal. I call this second property of the sensorimotor interaction that constitutes an experience of the outside world: “bodiliness.”

Finally, because there is generally a distance between sources in the world and our sensors, signals to the sensors can sometimes be interrupted when interfering objects interpose themselves between the sources and our sensors, and intercept the flow of information.^4^ Thus, a third characteristic that defines what it is to interact with the world is the implicit knowledge that information flow can on occasion be completely interrupted -- neural excitation can become quiescent and show no variability. We call this property of sensorimotor interactions that constitute exteriority: “interruptibility.”

Insubordinateness, bodiliness, and interruptibility^5^ are three “signature” attributes of the sensorimotor interactions that constitute what it is to be interacting with the outside world using our human sensory apparatus. Part of “what it’s like” to be having an experience with “exterior” qualities **consists in** implicitly being poised to note that the sensorimotor interaction has these properties. When these properties are lacking, then this will constitute what it **is** for an experience to be more “internal.”

Thus, consider **vision**. Retinal excitation changes radically when objects move in the visual field (insubordinateness). But excitation also changes radically when the eyes, the head, or the body move (bodiliness). Excitation is also dramatically altered when we blink or the light from the world coming into our eyes is interrupted by a passing object (interruptibility). This pattern of possibilities is the “signature” of what it consists in to be perceiving information residing in an external world. Vision provides an experience of exteriority.

Contrast this with the case when we are observing an afterimage. Here, because the afterimage is “stuck” to a given retinal location, moving the eyes or the body does not systematically modify the retinal activation in the way typical of external stimulation (no bodiliness). The spontaneous change in the afterimage is restricted essentially to fading and modulation by contrast and attentional effects (Wede and Francis, 2007) that are much more limited than the spontaneous displacements typical of normal vision (little insubordinateness). And the stimulation due to the afterimage is not interruptible by external events in the way that occurs for normal vision. In sum then, there is no longer the expected signature of exteriority, and after a moment of consideration of an afterimage, we attribute it to our “internal” world. The situation is similar to what occurs when one emerges sufficiently from dreams and hallucinations to be able to consciously consider the fact that when we move our body, the changes in incoming information do not follow the expected signatures of exteriority, and we realize we are not experiencing reality.^6^

In **audition**, sound intensity and spectrum as well as the temporal offset between the information registered in the two ears can vary when the sound source changes position (insubordinateness), but can also vary when the head or the body moves relative to the sound source (bodiliness). Sounds are also drastically modified when objects are interposed between the source and the ears (interruptibility). With this signature pattern, audition therefore also has an “exterior” quality. Exceptions to this occur for example in the case of tinnitus, and in the case of listening to headphones, where there is no effect of head movements on auditory spectrum and interaural delays. This type of dependency on body movements is what we designate as an internal, “inside the head” type of experience.

In **touch**, exploration of objects outside the body provides tactile stimulation that varies systematically as the hand moves (bodiliness), but can also vary systematically if the object moves (insubordinateness). The stimulation can cease when the object is removed (interruptibility). Again we have the signature pattern of exteriority. On the other hand in the case of clothing on one’s body, or if for example a vibrating stimulus is fixed to the skin, body movement does not appreciably change the stimulation received, and we no longer have the impression of information coming from outside in the world. Instead, such passive touch is localized on the body surface.

It is now interesting to consider **smell** and **taste**. Here the receptors involved do not have the property of measuring information at a distance, but on the contrary require actual contact of the source of stimulation with the receptor surface. As a consequence the receptor response is not dependent on body movement in the same signature way that is typical of vision, audition and touch. Moving the body (the nose, the mouth, and the tongue) has some effect on receptor activations: sniffing changes the intensity and distribution of molecules in different sensors; moving the tongue redistributes the food differently across the papillae. But there are not the same dramatic, fast, systematic, reversible give-and-take type changes that we can find in vision, audition and touch when the body moves back and forth, for example. Furthermore there is little spontaneous variation in smell and taste that occurs when no body movements are made, another indication that receptor input is not measuring something in the outside world.

These considerations therefore explain why smell and taste are not experienced as corresponding to, or localized in, the external world as is the case for vision, audition and touch. Smell and taste are more linked to the body, in particular to the nose and mouth, given that it is these body movements that do provoke change. Smell and taste are also more state-like, and have less of an ongoing quality because all the variability in receptor excitation must derive from voluntary active sniffing or tongue/mouth movements, and does not usually derive from spontaneous changes, nor can the flow be spontaneously quickly interrupted -- contrary to vision, audition and touch where the external world at a distance can change of its own accord or be interrupted by external events.

In the case of the **vestibular** sense, moving the body creates immediate changes in neural activation from semicircular canals and otoliths, since these measure acceleration of the head relative to the world. But contrary to vision, audition and touch, there is little spontaneous variation in this activation (and, a fortiori, no interruptibility), the only exception being when one is involuntarily subjected to external forces as in falling or travelling. This means that vestibular information is not experienced as being external, but rather as an “internal” state of balance or orientation.

Moving one’s limbs creates immediate **proprioceptive** feedback from muscle spindles, joint receptors and cutaneous information (bodiliness), but this feedback does not have the signature of exteroception, because there is no spontaneous variability (insubordinateness) of this feedback due to “outside” influences, nor can the flow of proprioception be interrupted. We therefore experience the location of our limbs as an internal state rather than as information coming from outside.

### 2.2. Metaphysical reminder

Let us not lose sight of what we are trying to do here. The article as a whole starts with the intuition that “locus” of experience and “imposingness” are fundamental components of why there is “something it’s like” to have an experience rather than “nothing it’s like.” The present section on “Bodiliness, insubordinateness, and interruptibility in external experiences” started by investigating the issue of “locus” and trying to explain why exteroceptive information gives us an “exterior” kind of sensation.

But has anything more been said in this section than the obvious fact that when we sense information in the outside world, it feels like it’s coming from the outside world? Yes, more has been said.

The point is to realize that, for the brain sitting inside the bony cavity of the skull, neural activity coming from the outside world is just like neural activity coming from inside the body or other brain areas. An explanation is needed for why some neural activity feels outside-worldly, some feels body-like, and some feels mind-like. There are two possible paths to answering this question.

The “classical” approach, corresponding to currently predominant views about the role of the brain in consciousness, would be to say that there must be something about the neural circuits involved in processing the different types of information that generates the different experienced loci of the feels.^7^ But this approach immediately faces the “hard problem” of explaining why and how certain neural activity generates an “exterior” feel, and another type generates another kind of feel: We are back to the “explanatory gap.”

The sensorimotor approach obviates the problem by saying that in order to understand why different feels have different experienced loci we should ask: What do we mean by this? For example, what does having an “exterior”-type feel **consist in**? And when we do this we realize that what we mean by having an “exterior”-type feel is that we are engaged in an activity where the degree to which we can modulate the experience through our voluntary actions has three “signature” properties, namely the properties of insubordinateness, bodiliness, and interruptibility. “Interior” and “mental” feels lack these properties in different ways. More work could be done to determine with greater precision the necessary and sufficient conditions for an experience to have an exterior, interior or mental quality, but here we shall take insubordinateness, bodiliness, and interruptibility as a first approximate signature.

### 2.3. Bodily experiences

**Hunger**, **thirst**, the need to **urinate** or **defecate**, the need to **breathe**, as well as **itches**, and **pain** correspond to fundamental needs of the organism and are sometimes called “primordial emotions” or “homeostatic emotions” (Craig, 2003; Denton, 2005; Damasio and Damasio, 2022). Part of what it is to have these experiences involves implicitly taking note of a variety of metabolic, physiological, and physical changes in our bodies that accompany the experiences (e.g., when hungry I may notice I have stomach cramps, when in pain I may notice that I start sweating and my heart beats faster). But these **accompanying** perceptual experiences can be dealt with in terms of the associated perceptual modalities: somatosensory, visual, auditory, etc. What fundamentally we want to explain is what is left after such accompanying perceptual components have been set aside, namely the underlying **essence** of the hunger, thirst, etc., experiences themselves.

Consider **hunger**. For the newborn baby, the essence of hunger involves a metabolic deficit that activates a collection of chemical, hormonal and neural states that, among other things, cause the baby to manifest its need in a variety of ways and cause it to eat food that is available. This ultimately results in remedying the metabolic deficit. Over the course of development the baby’s brain comes to learn the correlation between the collection of states signaling the metabolic deficit, the eating, and the return to homeostasis, thereby creating a motivation to eat when the appropriate states occur. I suggest that later in life, what we mean by feeling hungry consists in being engaged in implicitly mentally probing and verifying that currently the relevant collection of chemical, hormonal and neural states is activated that can potentially be modulated in a certain way by eating; by concomitantly implicitly noting the motivation to eat that accompanies these states; and also implicitly noting the accompanying somatosensory phenomena (the stomach cramps, lack of energy, etc).

But note that the effect of the bodily action of eating is unlike the effect of bodily actions in vision, audition and touch, where there can be immediate, reversible changes in neural activation (bodiliness). In the case of hunger, eating only has a much more long-term effect. Furthermore, the neural states underlying hunger do not vary quickly and spontaneously as a function of external influences as do the states underlying vision, audition and touch (insubordinateness). Finally the neural states underlying the hunger experience cannot be interrupted spontaneously by external events (interruptibility). For all these reasons therefore, the essential, non-somatosensory, aspect of hunger lacks the signature of exteriority and constitutes a more state-like, bodily, internal experience. Perhaps one could say that this non-somatosensory aspect is mainly just a motivation to eat.

Analogous points can be made about **thirst**, **urges to urinate**, to **empty the bowels**, and to **breathe**. In each case, having the experience involves a person being poised to make use of the fact that there is currently a set of neural and metabolic states that can be relieved by appropriate remedial body actions. But in all these cases the effect of the bodily actions is not reversible and immediate (no bodiliness), nor is it spontaneously altered from moment to moment (no insubordinateness) nor interruptible by external events (no interruptibility). Furthermore the experiences are all accompanied by concomitant perceptual phenomena that suggest bodily localizations. For example thirst is accompanied by a dryness of the throat; urges to breathe, urinate and defecate are associated with somatosensory experiences in the chest or lower body. For all these reasons the experiences do not have the signature of exteriority and are experienced as having an internal, bodily nature. The essential, non-somatosensory aspect of these experiences is probably purely motivational.

We can now turn to **pain**, **itches**, and **tickles**, where similar considerations apply, but where there is greater variability in the additional sensory components determining the location of stimulation on the body, the size or nature of the stimulation (pointed, blunt, rapid or slowly varying). These sensory components provide their own separate somatosensory experiences. As before, of interest to us here is the pain, itch or tickle *itself*, that is, the essential underlying components determining the particular kinds of aversive reactions and/or motivational changes. How do they depend on body movements? In certain cases of pain, itches and tickles imposed by outside stimulation, body movements can modulate these experiences in a more immediate and reversible way than compared to hunger, thirst, etc., where the effects of body movement are more long-term. Thus for pain, itches and tickles there is some bodiliness. Furthermore spontaneous variations in outside influences can rapidly modulate pain, itches and tickles (there is some insubordinateness). And pain, itches and tickles can be interrupted by the stimulus being removed (interruptibility). As a consequence we expect that pain, itches and tickles, when these are imposed by outside stimulation, can be experienced as stimulations impinging from the outside that are more ongoing, and less state-like than hunger, thirst, and the urges to breathe, urinate and defecate. On the other hand, particularly in the case of internal pains (for example headaches), the signature “bodiliness,” “insubordinateness” and “interruptibility” will be absent, and the experience will be taken to have an “internal” quality.

The affirmations in this section about the state-like character of bodily experiences are supported by everyday language. In English, French, and German, when we refer to bodily states, we use expressions that indicate a more state-like experience attributed to the body or the person as a whole: “I am hungry,” “I have a pain,” “I need to go to the toilet.” We do not say “I perceive my hunger” or “I sense the pain,” unless we mean that we are standing back and looking at our bodily states as external observers. This is in contrast to perceptual experiences where we say “I see the apple” or “I hear the violin,” and never anything like “I have a feeling of seeing an apple.” Interestingly one does say, in exploring with the hand: “I feel the apple in my hand,” or “I feel the softness of the feather,” but here the perception is of being (passively) touched, and as pointed out above, *passive* touch is indeed more state-like.

### 2.4. Emotions

Basic **emotions** like anger, sadness, fear, disgust, happiness and surprise, as well as secondary emotions like embarrassment, shame, excitement, amusement, etc., occur when externally perceived events and their mental interpretations lead to objective involuntary, genetically determined physiological, bodily and attentional changes over the short term or long term. There is therefore a perceptual process involved in triggering emotions, but the experience of the actual emotion is not itself perceptual. Rather, experiencing an emotion involves mentally taking note, manipulating, reviewing the facts, memories or reasoning that motivate one’s particular emotional state, as well as noting and probing and confirming the presence of certain physiological and bodily manifestations and motivational changes that characterize the emotion.

For example, one component of experiencing anger toward a person consists in the process of reflecting upon one’s mental relation to that person and on the things that the person has done or should have done, and noting that one is dissatisfied, annoyed, displeased, and that one has aggressive motivations toward that person; an additional component of anger consists in implicitly knowing that one is maintaining one’s posture, one’s facial expression, one’s jaw-set, one’s muscular tensions, etc. in states that correspond to the emotion of anger.

Does emotional experience involve the “signature” effects of sensory world-oriented experience? Clearly not, since making bodily movements does not alter the mental nor the neural states involved in the emotion in the “signature” way of creating immediate, systematic, reversible changes (bodiliness). Nor are there rapid, spontaneous fluctuations in the neural states that could be attributed to outside-world source (insubordinateness). Nor are emotions interruptible spontaneously by sudden external events (interruptibility). Emotions therefore are not experienced as having an outside-world, perceptual nature, but are taken to be states. Furthermore, the states are attributed to the person and their body as a whole, since they involve both bodily states and motivational and mental states: there are physical-body manifestations that can be probed,^8^ and there is the involvement of mental attitudes. The bodily state-like, whole-person aspect of emotions is corroborated by language use: We say “I feel sad,” “I am angry,” not “I perceive my sadness,” “I perceive my anger.”

### 2.5. Another metaphysical reminder

Section “Bodily experiences” and section “Emotions” have been concerned with showing how the subjective impression of a bodily, state-like quality associated with bodily experiences and emotions can be accounted for in terms of the fact that they lack the objective signatures of exteriority: they lack insubordinateness, bodiliness and interruptibility. Now again, as already stressed in section “Metaphysical reminder” for exteroceptive experiences, this claim is not a causal claim, but rather a **constitutive** claim: Careful consideration of what we **mean** when we say that bodily and emotional experiences have an interior, state-like quality is that we know implicitly that if we were to check the effect of moving our bodies, there would be little effect (no bodiliness); if we do not move our bodies, the spontaneous changes that we observe are minimal as compared to the situation with the perceptual senses (no insubordinateness) and that there is no immediate way the accompanying sensory flow can easily be interrupted (no interruptibility).

However, there is an additional, even more fundamental metaphysical point to be made. Underlying the account I am giving here of interiority or exteriority is the wager that by appealing to sensorimotor laws, a description of experiences can be given that accounts for **everything** that can be scientifically said about their qualities. No additional appeal to ineffable “qualia” is necessary to fully account for all aspects of experience. As a concrete example, I claim that careful examination of what a person **means** when they say they are experiencing hunger reveals that they as selves are mentally manipulating, noting, probing, or exploring certain changes in their motivation and potential ways of acting that are produced by certain physiological states of metabolic deficit. Importantly, it is NOT the case that the physiological states produce a feel of hunger, and that this in turn produces changes in motivation or ways of acting. On the contrary, the claim is that physiological states produce changes in motivation and ways of acting, and we **call** “hunger” the fact of being mentally engaged in noting these changes.

It will immediately be objected that this runs counter to the intuitive impression we all have of “really feeling” the hunger.^9^ Hunger, like all experiences, has a “felt,” impinging, “occurrent,” “ongoing,” “happening-to-me” quality that seems not to be explained by merely appealing to mental manipulation, probing etc. The claim of the sensorimotor theory that experiencing merely involves noting, probing, exploring our mental and physical dispositions seems to completely miss the fact that there is “something it’s like” to feel hungry. Feeling hungry is not simply a question of being currently mentally probing some dispositions.

But this is precisely where the purpose of the present article resides. The purpose is to show that if we bite the bullet and further ask ourselves what we might actually **mean** when we say that we “really feel” the hunger (or any experience), then it becomes clear that this apparently mysterious “something it’s like” actually does boil down to further implicit mental manipulation, probing and exploring: when we are experiencing, we as selves are implicitly engaged in noting and probing and exploring two very fundamental facts about our current state: the fact that what is happening to us is associated with a locus in the world, the body or the mind; and the fact that our current mental probing and exploring is partially taken over by what is happening, so that we partially lose control of our probing activity, and the experience has a certain degree of imposingness. My suggestion is that being implicitly aware of these two aspects of experience plausibly at least partially **constitutes** what people **mean** when they say there is “something it’s like” to have an experience. Future work may show that additional factors are involved, but these two account for a significant portion of what people mean.

The purpose of the sections up to here as well as the next section about mental experiences is to investigate the question of locus and show how it is linked to the control or lack of control that our bodies can exert over the flow of information. Section 3 on “Imposingness and loss of control of the body and attention” will then analyze in detail the question of imposingness and show that its objective counterpart in all types of experience corresponds to the loss in control over our voluntary body or attention deployment.

### 2.6. Mental experiences

**Mental experiences** like feeling confused, lonely, helpless, ridiculous, uncertain, puzzled, confident, resolved, well-disposed, patriotic, benevolent are, like emotions, triggered by the accumulation of perceptual information and its subsequent cognitive processing and interpretation. But this perceptual and cognitive processing does not constitute the mental experience itself, which rather involves mentally probing facts, memories and knowledge, as well as one’s resulting motivations, desires, beliefs and attitudes.

Are mental experiences perceptual and outside-worldly? Clearly not: they do not have the signature of exteriority. There is no direct, immediate, systematic, reversible effect of body movements on one’s mental experiences (bodiliness). There are no short-term outside-world influences that might create unpredictable fluctuations in one’s mental states (insubordinateness): Once established, the facts and memories at the root of the mental state do not fluctuate in the short term, and new probing will yield the same mental outcomes. There are no sudden interruptions of mental states due to outside influences (interruptibility).

As such therefore, mental states are even less susceptible to outside influences than state-like bodily experiences and emotions, and even less modified by body movements: this is essentially what we mean by being a “mental” experience. Although we do say “I feel confused,” “I feel lonely,” it may be more usual to say “I am confused,” “I am lonely,” suggesting an even closer association with the person and the mind, rather than the body.

### 2.7. Summary

The present Section 2 on “The external/internal/mental dimension of experience and control over information changes caused by voluntary body actions” has analyzed one of the two fundamental aspects of experience that I suggest contribute to why people say there is “something it’s like” to have an experience, namely the question of its locus and state-like nature: whether the experience corresponds to something occurring outside the body, inside or on the body in a state-like way, affecting the person as a whole, or of a mental kind. The claim is that **subjectively** experienced variations in the world/body/mind dimension of experiences correspond to **objective** variations in the degree of control that we can exert over changes in incoming information by means of voluntary body motions. Three aspects of control, namely bodiliness, insubordinateness, and interruptibility played a critical role as a “signature” of exteriority. When this signature is absent, the experience possesses either a more bodily, state-like quality that may affect the person as a whole, or a more mental quality.

It is important to again stress a metaphysical point. The claim is NOT that when sensorimotor interactions possess the signature of bodiliness, insubordinateness and interruptibility, an experience of exteriority is generated. To take this position would immediately raise the question of why this should be the case, and how such an “exteriority-feeling” could arise from particular laws of sensorimotor interaction.

Instead the claim is to say that **what we mean** by an experience having an exterior quality is that the underlying sensorimotor laws have the signature of insubordinateness, bodiliness, and interruptibility. Having an exterior quality **just is** having that signature. What we **mean** by being engaged in more bodily, state-like and mental experiences is to be noting that insubordinateness is reduced, bodiliness is much less immediate, and there is little interruptibility. Differences in quality of different types of perceptual, bodily, emotional and mental experiences are **constituted** by differences in the degrees to which these three “signature” laws apply.

The left-hand columns in Table 1 attempt to summarize briefly the objective analysis presented in this section, with the middle columns providing the corresponding subjective degree of exteriority and perceived locus of experience. Note that the entries in the table, as also the whole discussion presented in this section, are a first sketch subject to debate and more careful analysis. Nevertheless the work serves to demonstrate the potential of the approach that links objective facts about our ability to control sensory flow through our body movements, to the subjective impression we have that experiences have of “something it’s like.”

**Table 1:** Summary sketch of the conclusions in the article.

	OBJECTIVE			SUBJECTIVE		SUBJECTIVE	OBJECTIVE		
	How sensory flow depends on voluntary body movements						How voluntary control is lost		
	**INSUBORDINATENESS(can change without body moving)**	**BODILINESS(immediate dependence on body movements)**	**INTERRUPTIBILITY(can be interrupted)**	**Degree of exteriority**	**Locus of attribution**	**Type of presence or imposingness**	**LOSS OF CONTROL OF BODY**	**LOSS OF CONTROL OF ATTENTION**	**LOSS OF CONTROL OF MOTIVATION**
**Vision**	+++ visual input spontaneously changes all the time	+++ slightest eye/head movement changes input	+++ can be interrupted by blinking or occlusion	+++	World	Spatially extended, continual rich presence	+++ fast eye and head movements: orienting	+++ fast grabby alerting and orienting	− no automatic effect
**Audition**	+++ auditory input spontaneously changes all the time	+++ slightest body movement changes input	+++ can be interrupted by occlusion	+++	World	Spatially extended, continual textural presence	++ head movements: orienting	+++ fast grabby alerting and orienting	− no automatic effect
**Active touch**	++ explored objects can change spontaneously	+++ moving the hand changes what is touched	+++ interrupted by losing contact with objects	+++	World	Spatially extended but “latent” presence	+ minimal alerting and orienting	− minimal exogenous attention grabbing	− no automatic effect
**Passive touch**	++ less spontaneous changes on skin than for vision and audition	− moving body hardly changes passive touch	+++ can be interrupted by removing stimulation	−	Body surface	Spatially extended but “latent” presence	− little automatic alerting or orienting body movement	++ some exogenous alerting and orienting	− no automatic effect
**Taste**	− taste input doesn’t change spontaneously	+ only slight changes in taste from tongue movements	+ only slowly interruptible compared to other senses	−	In the mouth	Localized presence	− some automatic reactions to specific tastes (e.g., bitter)	+ alerting only from strong tastes	+ sometimes automatic effect on motivations to eat
**Smell**	+ smell input spontaneously changes only slowly compared to other senses	+ only slow changes in smell from moving or sniffing	+ smell is only slowly interruptible compared to other senses	+	Near the nose	Localized presence	− some orienting of smell direction	++ some orienting; some alerting from strong smelling	+ some automatic attractive or repulsive motivations
**Vestibular**	+ changes spontaneously only when whole body accelerated	+++ highly sensitive to any body acceleration	− not interruptible	−	Internal	Presence only for large accelerations	+++ automatic balance correction	+ alerting only from large accelerations	− no automatic effect
**Proprioception**	− no spontaneous changes: only changes when we move	+++ highly sensitive to any body movement	− not interruptible	−	Internal	No presence	+++ automatically controls movements	− no alerting or orienting	− no automatic effect
**hunger, thirst, urges to urinate, to empty the bowels, and to breathe**	− change only slowly	− only slow dependence on body movements	− not rapidly interruptible	−	Internal	Non-spatial imposingness	++ specific relieving actions	++ progressively alerting, preoccupying	+ Progressive urge to alleviate
**Pain, itches, tickles**	+ only sometimes quickly changes spontaneously	+ sometimes fast dependence on body movements	+ sometimes can be interrupted quickly	+	On the body	Non-spatial strong imposingness	+++ specific averting, retracting actions	+++ fast strong alerting, interrupting	+++ avoiding, alleviating
**Emotions: e.g., anger, sadness, fear, disgust embarrassment, shame, amusement.**	− do not very quickly change spontaneously	− are not affected quickly by body movements	− cannot be quickly interrupted	−	Internal	Imposingness on body and whole self	++ specific characteristic social body signaling	++ specific diffuse modulating, guiding effects	++ specific modulating, guiding effects
**Mental experiences: e.g., confused, lonely, helpless, ridiculous, puzzled, confident, benevolent**	− do not change spontaneously from external causes	− not directly affected by body movements	− cannot be quickly interrupted	−	In the mind	Imposingness on mind	− no automatic bodily manifestations	− no exogenous take-over of attention	− motivations rationally not exogenously determined

For each type of experience shown in column 1, the three OBJECTIVE columns on the left give the objective dependence of sensorimotor flow on body movements. The three OBJECTIVE columns on the right give the extent of objective loss of control over body movements, attention and motivation. The middle SUBJECTIVE columns give the subjective impressions of exteriority, the locus of the experience and the type of presence or imposingness constituted by the OBJECTIVE facts. Note that these are very approximate summaries– more detail is given in the text. Many of the entries may require revision and discussion. The purpose is essentially to show that the approach is a promising way to link OBJECTIVE facts about loss of control with SUBJECTIVE facts about locus of attribution and imposingness that may be fundamental factors contributing to the fact that people claim that experiences have « something it’s like ».

The right-hand columns in the table summarize the results of the next section of the article (Section 3 on “Imposingness and loss of control of the body and attention”) concerning the second fundamental contributor to “something it’s like,” namely “imposingness,” and its objective correlate.

## 3. Imposingness and loss of control of the body and attention

Section 2 on “The external/internal/mental dimension of experience and control over information changes caused by voluntary body actions” gave an objective account of why experiences are attributed to different loci: the world, the body and the mind, thereby explaining one important aspect of “what it’s like” to have an experience. A second very fundamental aspect of “what it’s like” is the fact that experiences can “possess us”: they can “grip” us, inhabit us, or “impose themselves” on us: they can force us to take notice of them, they can seem phenomenally “present” to us. I shall use the general term “imposingness” to cover all these **subjective** aspects of the “what it’s like” of experience.

An **objective** fact about brain functioning is at the root of this subjective impression of imposingness: It is the fact that many brain systems are equipped with hard-wired mechanisms that function independently of our will, and can exogenously take over different functions in different ways. They can take over **voluntary bodily** functions, and/or our **attention** and/or our **motivations**: we partially **lose control** over our bodies, our voluntary actions and motivations and thereby our cognitive processing. Loss of control caused by these mechanisms is at the root of the imposingness of many types of experience and makes a second major contribution to “what it’s like” to have an experience.

The following subsections will look in detail at different types of experiences and the different ways exogenous attention-grabbing mechanisms determine different aspects of imposingness. In particular, we will see that the exogenous mechanisms can have two kinds of effects: a purely **alerting** effect, impeding current processing; or they can additionally be **orienting** and assisting. This latter kind of effect is found essentially in vision and audition and gives these experiences a particular “spatio-temporal” kind of presence.

### 3.1. Loss of control in perceptual experiences

All five of the classic sense modalities of seeing, hearing, touching, tasting and smelling are equipped with genetically hard-wired **alerting reflexes** that register sudden large changes in sensory signals, and that exogenously capture our attention and **interrupt** current motor activity and cognitive processing. Thus, sudden large flashes of light, loud sounds, tactile impacts, pungent smells and tastes can alert us and cause us to “freeze,” ceasing our current perceptuo-motor exploratory activities and heightening our attentional availability. As a consequence, we are implicitly aware that our current motor, attentional and cognitive activity might at any moment suddenly be interrupted and taken over. This fact contributes to giving the five classic sense modalities a kind of precarity, immediacy, acuteness or “realness” -- relating to the fact that the possibility of such interruptions show the existence of external causes over which we have no control.

In addition to **impeding or interrupting** ongoing perceptuo-motor exploratory processing in the case of very large sudden changes, the alerting reflexes can in some cases be accompanied by automatic **orienting** mechanisms that **assist** rather than impede ongoing processing.^10^ I illustrate below how, particularly for vision and audition, these orienting mechanisms contribute an additional component to the “what it’s like” of the associated experiences: in addition to the immediacy, acuteness and realness provided by the alerting reflexes, the orienting mechanisms provide an impression of “spatio-temporal presence” that make vision and audition appear to us as having an external “continually-displayed-before-us” quality.

Thus, in **vision** there are channels selective to localized spatio-temporal transients -- small, localized signals in peripheral vision like motion cues or sudden onsets and offsets -- that attract involuntary eye saccades and orient attention in an automatic, exogenous, involuntary fashion. Even in the absence of temporal changes in retinal stimulation, saccades and attention tend to move rapidly and involuntarily toward information-rich, salient, small, high contrast areas -- for example corners and isolated spots (*cf.* review by Carrasco, 2011). Instead of **interfering with** processing as is the case for global alerting reflexes, these attentional and saccadic orienting mechanisms promote registration of interesting new material and thereby **assist** processing. The slightest small change in the peripheral visual field immediately exogenously attracts attention, and processing of the new event is initiated or enhanced. Because this surveillance mechanism immediately alerts us to the slightest new event, we have the illusion that our visual field is **continually spatio-temporally phenomenally present** in infinite detail, despite the fact that our peripheral vision is actually severely limited in its spatial and color resolution (O’Regan, 1992). The phenomenon of having the erroneous impression of seeing everything in rich detail has sometimes been called the “grand illusion” (Noë and O’Regan, 2000), and is instantiated in change blindness (*cf.* review by Simons and Rensink, 2005).

**Audition** involves a very different type of receptor as compared to vision. Estimation of the direction and distance of sound sources over a wide, spatially extended area are achieved by sophisticated temporal processing of the information from the two ears making use of reflections in the environment and the convolutions of the outer ear lobes. However because spatial resolution is much worse as compared to foveal vision, the “auditory scene” is more of an extended texture than a distribution of individuated auditory objects.

Within this context an **orienting mechanism** operates similar to the one found in vision: in this, salient auditory events solicit attention and/or head orienting (and in animals, ear-orienting), which contributes to improve processing (Spence and Driver, 1994; Diaconescu et al., 2011). However, because head movements are slower than eye movements, and because of the lesser spatial resolution, the illusion of “continual spatio-temporal presence” is less pronounced as compared to vision. We may have the impression of a continually present and surrounding sound texture, but the individual sound sources do not appear to be simultaneously phenomenally “present” as they do in vision: the different instruments in the orchestra playing before me merge into a single sound texture and require attentional resources to be individuated.

In conclusion, for vision and audition: in addition to the precarity, immediacy, acuteness or “realness” produced by the presence of alerting reflexes, these modalities have the peculiarity that they give us an impression of an extended, spatio-temporally continually present “scene,” displayed in front of us. This aspect of the “what-it’s like” of vision and audition can be accounted for in terms of the genetically hard-wired orienting mechanisms that assist attentional and cognitive processing for these modalities.

The automatic reflexes for **passive touch** are more of the “alerting” than the “orienting” kind. A sudden tapping on one’s body may alert one’s attention immediately but no bodily movement can contribute to improve the processing or extraction of information at that location. Perhaps orienting attention to the location of the tapping can help further processing, but the effect is not comparable to the mechanisms found in vision and audition. For that reason our impression of touch on our bodies is **not** of continual phenomenal spatio-temporal presence. In order to feel the softness of my socks on my feet, I must voluntarily cast my attention upon them. There is nothing like an ongoing, phenomenally currently experienced, spatially extended “field of passive touch.”

The situation is even worse for **active touch** because the hand, usually used to explore actively, has a very limited spatial extent. Since, as for passive touch, there is no equivalent of the attentional and bodily orienting mechanism that we have for vision and audition, the “field of active touch” cannot have the same degree of spatio-temporal presence that there is for vision and audition. The impression of our tactile environment must be constructed cognitively, by accumulating successive slow palpations. We could say that our impression of phenomenal presence is “latent” rather than “actual.” This is in contrast to vision, where we have the impression of continually seeing all the details of everything displayed before us.

For **taste and smell**, automatic, involuntary body motions would seem to be restricted to the **alerting responses** corresponding to aversive reactions to extreme tastes and smells.^11^ This kind of mechanism does not contribute appreciably to guide further processing of those events. As a result, taste and smell do not provide a subjective impression of continual spatio-temporal presence analogous to what we have for vision and audition. Our taste and smell “worlds” are only phenomenally “present” to us when we are alerted to a change or when we voluntarily cast our attention on them (this point has been made for smell by Sela and Sebel, 2010).

When we suffer sudden changes in orientation or accelerations as when losing our balance or travelling in a vehicle, changes in **vestibular** input can provoke rapid automatic righting reflexes that can also grab attention. There is therefore loss in control over body motions and over attention in the short term, meaning that the vestibular sense is experienced as somewhat bodily imposing. Note however that most of the time, the vestibular modality serves in an automatic mode to keep our balance, producing involuntary adjustments to our posture. But unlike vision where oculomotor reflexes contribute to guiding further cognitive processing, in the case of vestibular input, these involuntary adjustments do not capture our attention to guide cognitive processing. Thus the “sense of balance” function of vestibular processes has no imposingness, and is therefore not experienced as being spatio-temporally present to us. Only when we are subjected to external movements or loose our balance do we perceive the world or our bodies as spatially “present” and moving relative to each other. The considerations in Section 2 on “The external/internal/mental dimension of experience and control over information changes caused by voluntary body actions” related to control of information flow further contribute to explaining why vestibular information is not experienced as a perceptual sense, but as more state-like or mental.

Similar to the “sense of balance” aspect of vestibular processes, **proprioception** seems not to be equipped with automatic mechanisms to obstruct voluntary movement or to grab attention. Certainly proprioception intervenes continually and involuntarily in low-level control loops to modify moment-to-moment control of body motions, but this does not interfere with one’s voluntary control of movements, nor does it prevent normal attentional processes. As a consequence proprioception is not imposing. Section 2 on “The external/internal/mental dimension of experience and control over information changes caused by voluntary body actions” also suggests that proprioception should not be considered a form of perception, but more state-like and as a form of knowledge. The two considerations together explain why proprioception, like the vestibular sense, is rarely considered a sense modality on a par with vision, audition, touch, taste and smell.

An interesting point can be made about the difference between the perceptual presence of the five classic senses of seeing, hearing, touching, tasting and smelling, and the lack of such presence for sense of balance and proprioception. The suggestion here is that the difference is due to a lack in the ability of sense of balance and proprioceptive signals to interrupt and re-orient cognitive processes. It would be very exciting to be able to demonstrate anatomically that the five classic senses possess some kind of direct access to (presumably prefrontal) cortical areas that can interrupt cognitive processing there, whereas sense of balance and proprioception do not have such “interrupt” connections to areas dealing with cognitive processing.

### 3.2. Loss of control in bodily experiences

This section considers homeostatic bodily experiences like hunger, thirst, drowsiness, fatigue, the need to urinate or defecate, to breathe, itches, tickles, and pain. As done in Section 2 on “The external/internal/mental dimension of experience and control over information changes caused by voluntary body actions,” accompanying perceptual aspects of these experience are left aside, since they can be accounted for in terms of the appropriate perceptual modality. It will be seen that what determines the imposingness of the bodily states **themselves** is the different ways in which these bodily experiences make us lose voluntary control over our body movements, of our deployment of attention, and our motivations.

Concerning voluntary body movements, in the case of fatigue, drowsiness, and pain, our voluntary physical actions are impeded by being slowed or modified. This may also occur in cases of extreme hunger and thirst. In the case of needs to urinate, defecate and breathe, as well as for itches, tickles and pain, there are automatic, involuntary movements or avoidance reactions that interfere with our normal voluntary motor control. In all bodily experiences there is therefore some kind of loss of control over voluntary body movements that determines part of its “what it’s like.”

Control of attention can also be appreciably lost for all these experiences. Depending on the intensity of the experience, attention will be more or less strongly, involuntarily and automatically deviated from other activities and will focus on the task of taking active steps that allow restoration of bodily homeostasis. This “grabbiness” of attention corresponds to a loss in control over attentional deployment that may be another aspect of the bodily “what it’s like” of these experiences.

Concerning motivation, finally and most obviously, all of the homeostatic bodily experiences impose an automatic, involuntary, strong motivation to alleviate and satisfy the underlying need by taking appropriate actions either in the short term (need to breathe, itches, tickles, and acute pain) or in the medium and long term (hunger, thirst, fatigue, drowsiness, and chronic pain). This type of involuntary, imperious motivation can interfere with other ongoing activities. It constitutes a third way control is lost, also contributing to the subjective bodily imposingness that constitutes the “what it’s like” of bodily experiences.

In conclusion, for bodily experiences, there can be loss of control over movements, over attention and over motivation. These three sources of loss of control may partially constitute what people fundamentally mean when they say that these experiences have a strong bodily imposing “what it’s like.”

As in Section 2 on “The external/internal/mental dimension of experience and control over information changes caused by voluntary body actions,” note the metaphysical foundation of the argument. The claim is not that bodily experiences **produce** the accompanying loss in control in body, attention and motivation. The claim is that the imposing “what it’s like” of these experiences partially **consists in** the fact that they cause us to lose control over body, attention and motivation.

### 3.3. Loss of control in emotions

Like the bodily experiences discussed above, the “basic emotions” of anger, sadness, fear, disgust, happiness and surprise are accompanied by metabolic, physiological and physical changes in the body that we may perceptually note when we have emotions. But setting aside these perceptual manifestations, of interest for the underlying essential “what it’s like” of emotions is again the loss of control that emotions involve over our potential voluntary physical, attentional and mental actions.

Concerning body movements, as for the bodily experiences, in the case of basic emotions our movements may be involuntarily globally slowed (sadness) or speeded (anger, happiness). But additionally, involuntary changes in body posture and facial gestures may occur whose function is presumably social signaling. Because these changes are involuntary, they interfere with, and cause us to partially lose control of our normal body movements. Each different emotion is associated with specific types of bodily manifestations, causing a different kind of interference and giving each emotion a somewhat different “what it’s like.” But globally all the basic emotions involve loss in control of certain bodily actions, and so provide a “bodily” “what it’s like.” The effects are different from those found in bodily experiences because the involuntary overt bodily changes (postures, facial gestures) play a role in social signaling. The “what it’s like” involved has not only a bodily, but also a social nature, linked to the person’s relation to their social context.

Concerning attention, as was the case for bodily experiences, attentional deployment is strongly affected in the case of basic emotions. But here there is a slight difference with bodily experiences. In bodily experiences, attention is of an alerting nature, interfering with other activities and orienting cognitive processing toward the motivation of satisfying the underlying physiological urge (to eat, drink, breathe, avoid the pain, etc). In the case of the basic emotions the effect on attention is also alerting, but the task to be accomplished is less precisely defined. One could say that attention occupies the person in a more general, less specific way. The imposingness seizes one more globally, modulating one’s approach to all of one’s activities in a general way. The “what it’s like” of emotions “moves” one personally, in addition to bodily.

Concerning motivation, unlike bodily experiences, the motivations involved in emotions are less precise and more ill-defined than those for bodily experiences. The loss in control over one’s motivation is more diffuse, affecting one’s mental states, causing one to think in a different way. This adds a “mental” aspect to the “what it’s like” of emotions.

In conclusion, for basic emotions, the loss in control over the body involves overt body movements that affect social signaling. The interference with attention deployment and motivation is more diffuse, more global, and less precisely defined than in the bodily experiences. The “what it’s like” of emotions as a consequence involves a gripping of the body in a general way, but also affects one’s social self as well as mentally. Emotions “move” one not just bodily but also personally, socially and mentally.

The above discussion on basic emotions can be extended to secondary emotions like embarrassment, shame, excitement, amusement, satisfaction, relief, guilt and pride, etc. Here there are also involuntary bodily manifestations, although these are not as clear-cut as for the basic emotions. Perhaps some (like flushing from embarrassment, breathing a sigh of relief, etc) have genetic components, and others may be culturally determined. What is important for their “what it’s like” is that these bodily phenomena are involuntary, and so make us partially lose control over our bodily movements. Attention deployment can sometimes be involuntarily affected (embarrassment, excitement, and guilt) and motivations are modified, but not so much in an automatic, involuntary way: instead changes in motivation are the product of mental states and are only involuntary because their complexity is such that we have difficulty voluntarily controlling them.

In sum, emotions involve specific, involuntary bodily manifestations that have a social signaling role. Additionally, emotions create changes in bodily attitudes and motivational states. Compared to bodily states, emotions, with their strong sensory, mental, bodily and motivational grip, effective in the short term and the long term, have a “what it’s like” that is experienced as affecting us in a sensory, social, and mental fashion. One could say that we are affected more as a whole person rather than just physically as is the case with bodily experiences.

### 3.4. Loss of control in mental experiences

There is a vast variety of what we shall call “mental experiences” like feeling lonely, helpless, confused, uncertain, familiar with something, patriotic, benevolent, bored, suspicious, confident, doubtful. How are they different from bodily states and emotions?

Continuing our logic of investigating the loss of control of the body, attention, and motivation in determining “what it’s like,” we can see the following.

As concerns the body: There are of course bodily consequences from the perceptual, attentional and mental processes that trigger these mental experiences, but they constitute attitudes, dispositions or motivations determining how we act in the medium- to long term. They are not genetically pre-wired dispositions like in the bodily states of hunger, thirst, etc. They are not immediate innate bodily changes that have a role as social signals. Rather, they are complex behavioral dispositions resulting from cognitive processing and social functioning. There is thus **no loss in control of the body** as there was for bodily experiences and emotions.

As expected therefore, subjectively, the imposingness of mental experiences does not have a bodily nature, and is experienced as mental, directly determining mental attitudes rather than bodily attitudes (although mental experiences do affect bodily attitudes indirectly).

Concerning attention: Experiencing a mental state like loneliness involves probing, with one’s attention and cognitive processing, the various ideas, memories, facts, dispositions that constitute what the experience of being lonely involves. These ideas, memories, etc., are entirely mental things and are under our voluntary mental control. There is no outside bodily or sensory influence that might involuntarily modify them as is the case in bodily experiences and emotions. There can be no sudden, uncontrollable event that interrupts this probing process and grips attention. There is therefore **no loss in control over attention**: no phasic takeover of attentional processes in mental experience.

Concerning motivation: mental experiences involve particular motivations to do things, but these motivations are entirely the result of one’s own voluntary mental actions. There is **no loss in control of one’s motivations** caused by genetically pre-wired mechanisms as in the case of bodily experiences or basic emotions.

All these facts showing no loss in control in the case of mental states have the consequence that unlike bodily states and emotions, which seem phenomenally “present” to us through their imposingness, mental states do not have this phasic imposingness. In order to be experienced, mental states have to be probed through a voluntary attentional effort. One only experiences one’s loneliness when one stands back mentally and reflects upon the fact of having the experience. It is not something that is uncontrollably, phenomenally present over time like hunger or fear.

As a result, mental experiences are not perceived as being “phenomenally present.” Their “what it’s like” is of an entirely mental type.

## 4. Conclusion

The present article accounts for two **subjective** aspects of “what it’s like” to have an experience: the degree to which experiences have **external, internal, or mental** qualities, and their phenomenal **imposingness** or spatio-temporal **presence**.

The two subjective aspects are seen to be **defined by** (or to **consist in**) two **objective** facts about what we do when we are having an experience. The first fact concerns our **control over the flow of sensorimotor information** that underlies the active bodily or mental probing that constitutes having an experience. The second fact concerns the effects of automatic, involuntary, hard-wired mechanisms that make us partly **lose voluntary control** over our body and over the deployment of attention and cognitive processing. Both types of control are objective, third person measurable aspects of the ongoing active (mental or physical) probing and exploring that implicitly underlies all experiences.

The suggestion is that locus and imposingness are important fundamental contributors to the fact that people say that there is “something it’s like” to have an experience: the fact that people use terms like “felt,” ongoing, occurrent, happening to me, “present,” real, etc., to describe experiences. Future work may show that additional contributors may exist, but these two seem to represent a first sketch that account for many aspects of what people say.

Previous work had already started to approach other aspects of subjective feels that are often considered mysterious, like the intra-modal differences question of why, for example, red seems red rather than green; or like the inter-modal differences question of why, for example, vision seems visual rather than auditory (for an overview see O’Regan, 2010). The present work adds to this a contribution to the more general question of why experiences have “something it’s like” at all.

Taken together, the work shows that the active sensorimotor constituent of experiences constitutes an additional source of explanation over and above the explanations used in classical accounts of phenomenal qualities. This enables the sensorimotor approach to make useful steps toward a scientific account of the “hard” problem of the “what it’s like” of bodily, emotional, sensory and mental experiences. The method consists in “dividing and describing”: dividing up the different aspects of what people mean when they say experiences have “something it’s like,” and describing these different aspects in terms of the mental probing and sensorimotor interactions that people are engaged in and that define what having these experiences **consists in**.

Note that the suggestions in this article are only preliminary sketches designed to demonstrate the feasibility of this approach. Further work is necessary using objective psychophysical methods to verify the claims made here about the effects of body movements on the information channels corresponding to the different experiences treated here, and about the movement-hindering, attention-grabbing, and motivation-altering capacities that underlie them. Further work investigating the connectivity of specific attention-grabbing neuro-anatomical pathways may also buttress the present approach.

## Author contributions

The author confirms being the sole contributor of this work and has approved it for publication.

## Funding

Financing was partially provided by European FET Grant 952095 IM-TWIN, Université Paris Cité and Planet Learning Institute.

## Conflict of interest

The author declares that the research was conducted in the absence of any commercial or financial relationships that could be construed as a potential conflict of interest.

## Publisher’s note

All claims expressed in this article are solely those of the authors and do not necessarily represent those of their affiliated organizations, or those of the publisher, the editors and the reviewers. Any product that may be evaluated in this article, or claim that may be made by its manufacturer, is not guaranteed or endorsed by the publisher.

## Footnotes

^1^Whereas in mainstream philosophy the notion of cognitive access will be construed as implicitly involving the creation of internal representations, my own use of the term would avoid the concept of representation, which, as noted, e.g., Myin and Loughlin (2018) is an "unexplained explainer."

^2^Clark (1996) admirably analyses how psychophysical methods can be used to describe such "quality spaces." But while Palmer (1999) suggests that an explanation for their structure may be out of the bounds of science, the sensorimotor account suggests that implicit sensorimotor laws may provide a scientific source of explanation (e.g., for color see Philipona and O’Regan, 2006; Vazquez-Corral et al., 2012).

^3^The sensorimotor approach assumes that the self is a necessary part of what it is to be conscious (*cf.* e.g., O’Regan, 2011, 2012), since under the usual definition of the notion of "experiencing," when people consider themselves to be having an experience, they are implicitly, prereflectively (but also sometimes reflectively) attributing the experience to *themselves*. Treating this issue very carefully, Kriegel and Zahavi (2016) and Nida-Rümelin (2017), for example, have come to the same conclusion.

^4^An exception to interruptibility could occur in the cases of senses like the sense of gravity and electric or magnetic senses in animals, where bodies interposed between the source and the sensor only have a weak effect or no effect at all.

^5^The concepts of bodiliness and insubordinateness were previously suggested in O’Regan et al. (2004, 2005), in addition to "grabbiness," which will be mentioned here in Section 3. Interruptibility is a notion alluded to in a study on distal attribution (Auvray et al., 2005).

^6^Of course at first in dreams and hallucinations, before one does such conscious probing, one has the illusion of external reality. Sensorimotor theory accounts for dreams and illusions by saying that either they involve having incorrect beliefs about sensorimotor laws or they involve correct beliefs but incorrect registration (through drugs or cortical activation) of sensorimotor laws.

^7^This idea is a modern variant of Johannes Müller’s notion of "specific nerve energy." For an interesting discussion see Rachlin (2005).

^8^Nummenmaa et al. (2014) for example give bodily localizations of the feel of different emotions.

^9^The difference with the Jamesian, behaviorist idea that "you do not run because you are afraid, you are afraid because you run," is that the sensorimotor theory adds an essential cognitive component (you are mentally probing etc); the sensorimotor theory also emphasizes dispositions rather than necessary actions; and the sensorimotor account also accounts for the "felt," "something it’s like" aspect of experience, as explained in the present article.

^10^Previous work has used the term "grabbiness" or "alerting capacity" to refer indiscriminately to both the alerting and the assisting kinds of mechanisms (O’Regan et al., 2004, 2005).

^11^Although there may be some slight orienting reflex for smell allowing for scent following (*cf.* Porter et al., 2007).
